# Haloperidol-Induced Dystonia due to Sedation for Upper Gastrointestinal Endoscopy: A Pediatric Case Report

**DOI:** 10.1155/2019/3591258

**Published:** 2019-03-27

**Authors:** Kazufumi Yaginuma, Masahiro Watanabe, Kyohei Miyazaki, Atsushi Ono, Hiromichi Murai, Maki Nodera, Yuichi Suzuki, Kazuhide Suyama, Yukihiko Kawasaki, Mitsuaki Hosoya

**Affiliations:** ^1^Department of Pediatrics, Shirakawa Kosei General Hospital, Fukushima, Japan; ^2^Department of Pediatrics, Fukushima Medical University, Fukushima, Japan

## Abstract

Dystonia is a movement disorder characterized by sustained muscle tone. Antipsychotic agents sometimes cause acute dystonia that can rapidly worsen within a few hours or days. Because healthy children rarely receive antipsychotic agents, it is unusual to see antipsychotic agent-induced dystonia in pediatric emergency departments. We report a rare case of a 12-year-old healthy boy who presented with acute dystonia after administration of haloperidol for sedation. He was suspected of laryngeal dystonia because stridor and desaturation were present. The symptoms disappeared with the administration of hydroxyzine. Rapid diagnosis was important in this case because laryngeal dystonia is a potential life-threatening complication due to upper airway obstruction. Considering the risk of side effects, doctors who are not accustomed to administering pediatric anesthesia should consult a pediatrician and/or an anesthesiologist prior to administration of anesthetics to pediatric patients.

## 1. Introduction

Acute dystonia is a movement disorder characterized by sustained muscle tone that can rapidly worsen within a few hours or days. It typically occurs in the head, neck, and trunk muscles as torticollis and/or opisthotonus [1]. When the disorder involves the larynx, it could be life-threatening because it results in upper airway obstruction. Therefore, rapid diagnosis and treatment are important [2, 3]. Medications, particularly the antipsychotics, such as dopamine receptor antagonists, cause acute dystonia in some cases. Drug-induced dystonia is a well-known adverse event in adults. However, it is an uncommon clinical condition for children because healthy children rarely receive such medications. Therefore, that children have drug-induced dystonia in pediatric emergency department is a rare possibility. Herein, we report a rare case of a 12-year-old boy who presented with acute dystonia after administration of haloperidol for sedation.

## 2. Case Presentation

A 12-year-old boy, with body weight 39 kg, presented with eye deviation, trismus, and hypertonia of the limb, and was admitted to the pediatric emergency department. He had undergone upper gastrointestinal endoscopy 1 day prior as a regular examination for duodenal ulcer. He had been administered haloperidol (total 4.5 mg) intravenously for sedation because he had exhibited a drug rash suspected to be a reaction to previously administered midazolam. His symptom developed during exercise about 24 hours after the administration of haloperidol. He had not been administered antipsychotics, including haloperidol and antiemetics, previously. He had no history of drug abuse or alcohol intake. He had no known allergy.

In the pediatric emergency department, his vital signs were within the normal ranges for his age. Eye position repeatedly showed bilateral left or upward deviation. He exhibited trismus, left deviation of the lip, lip smacking, puckering and pursing, jaw swinging and chewing, torticollis, opisthotonus, hypertonia of the limb, and action tremor. The symptoms were observed while he was awake but disappeared when he was asleep.

The results of the laboratory tests were normal, including white blood cell count (4200/*μ*L; 4000-10700/*μ*L), hemoglobin (13 g/dL; 12.2-15.7 g/dL), C-reactive protein (0.06 mg/dL; <0.15 mg/dL), aspartate aminotransferase (26 IU/L; 15-31 IU/L), alanine aminotransferase (14 IU/L; 9-32 IU/L), blood urea nitrogen (11 mg/dL; 6.8-19.2 mg/dL), creatinine (0.51 mg/dL; 0.39-0.62 mg/dL), serum sodium (141 mEq/L; 138-144 mEq/L), serum potassium (4.6 mEq/L; 3.6-4.7 mEq/L), serum calcium (9.8 mg/dL; 8.7-10.1 mg/dL), and creatine kinase (170 IU/L; 62-282 IU/L). Analysis of cerebrospinal fluid revealed it to be clear in appearance, with a cell count of 1/*μ*L, and normal glucose and protein levels (68 and 24 mg/dL, respectively). Cranial computed tomography, and magnetic resonance imaging showed no abnormalities. Blood concentration of haloperidol, while at the emergency department (about 24 hours after administration), measured using enzyme immunoassay method was 2.8 ng/mL (therapeutically effective concentration range is 3.0-17.0 ng/mL).

Although the diagnosis was not clear at that time, we transferred the patient to a tertiary care institution, as he additionally showed stridor and desaturation. The pediatric neurologists performed examinations, including electroencephalogram analysis. Paroxysm was not indicated and neither was epilepsy. Because the symptoms had appeared only when the patient was awake, he was diagnosed with acute dystonia due to haloperidol. Within a few minutes of administration of hydroxyzine, he fell asleep and was symptom-free, and he did not require intubation. Subsequently, he was symptom-free and was discharged after 3 days. One week later, he attended a follow-up visit and showed no symptom recurrence.

## 3. Discussion

Derinoz and colleagues reported that 4 of 55 patients (7.3%) with drug-induced dystonia developed laryngeal dystonia in the pediatric emergency department [8]. In the present case, the patient appeared to have developed laryngeal dystonia because stridor and desaturation were present.  Table 1 shows previous pediatric cases of laryngeal dystonia due to administration of antipsychotics [4–7]. Most of the patients were their late teens, thus the present case describes the youngest patient. Most of the patients had self-administered the antipsychotics; there have been no cases with antipsychotic administration for sedation as in the present case.

The underlying mechanism of acute dystonia is not fully understood. However, it is believed that as nigrostriatal D_2_ receptors play an important role in the initiation and control of movement, when an antipsychotic drug blocks the D_2_ receptors in the basal ganglia, such as in the caudate nucleus, putamen, globus pallidus, and substantia nigra, acetylcholine release predominates in the striatum, and results in extrapyramidal disorders [9, 10].

In the present case, other extrapyramidal symptoms complicated the diagnosis; these included repetitive involuntary movements of the mouth and lip, as well as action tremor. The other diagnoses considered were electrolyte imbalance, partial seizure, encephalitis, toxicosis, simulation, and conversion. Electrolytes were in the normal range. Electroencephalogram showed no evidence of partial seizure. Cerebrospinal fluid examination, cranial computed tomography and magnetic resonance imaging also showed no evidence of encephalitis. There was no history of illicit drug intake, neurally mediated syncope, psychosomatic disease and contact with poison. Simulation and conversion could not completely negated, however these were considered negative because he had no such histories and no signs were observed latter.

Antipsychotics and antiemetics are frequent causes of drug-induced dystonia [8, 11], which is often observed in teenaged males [12, 13], with the risk decreasing with age [14]. For the patient in this case, haloperidol was used for sedation; however, blood concentration at emergency department was not high. Although acute dystonia develops in a dose-dependent manner, it is not related to blood concentration. It is thought that high intracerebral concentration affects onset because haloperidol easily traverses the blood brain barrier due to its high lipid solubility. Magliozzi and colleagues reported that drug-induced dystonia can occur at low blood concentrations of haloperidol.

Recent use of cocaine, previous occurrence of acute dystonia, younger age, male sex, and use of high doses of antipsychotic are important risk factors for the development acute dystonia [14]. Younger patients are more susceptible to the development of drug-induced dystonia because D_2_ receptor activity reduces with age [15, 16]. Although the pathology is unclear, males are more likely to develop drug-induced dystonia than females [12, 13]. Our patient was at a high risk of acute dystonia because the last three risk factors were relevant to his case.

Antihistamines, biperiden, and benzodiazepine are effective for the treatment of drug-induced dystonia [8, 11, 16]. The symptoms improved immediately with administration of hydroxyzine in this case.

Benzodiazepine alone or in combination with additional opioids is commonly used for sedation during gastrointestinal endoscopy in adults [17]. In children, midazolam alone has shown limited efficacy and safety [18]. Several studies suggested that midazolam and/or propofol combined with additional opioids is suitable and safe for sedation of children [19–24]. The patient in this case had been administered haloperidol, thereby avoiding benzodiazepines, because he had history of a drug rash possibly related to midazolam. However, considering the risk of side effects, the gastroenterologist should have consulted a pediatrician and/or an anesthesiologist.

We have reported a case in which a 12-year-old boy presented with acute dystonia due to administration of haloperidol for sedation. Because acute dystonia could cause airway obstruction, rapid diagnosis is important. Doctors who are not accustomed to administering pediatric anesthesia should consult a pediatrician and/or anesthesiologist prior to administration of anesthetics to pediatric patients.

## Figures and Tables

**Table 1:** Summary of all the reported cases of antipsychotic-induced laryngeal dystonia in pediatric patients.

Study Author	Age	Sex	Agent	Time to onset from ingestion of antipsychotics	Treatment	Clinical Course
Goga et al. [4]	16	female	PO Aripiprazole	6 days	IM benztropine	Resolved rapidly with treatment
					PO diazepam	
Russell et al. [5]	16	male	PO Chlorpromazine	2 days	diphenhydramine	Resolved rapidly with treatment
Kanburoglu et al. [6]	14	female	PO Chlorpromazine	NA	IV diphenhydramine	Resolved in 10 min with treatment
					IM biperiden	
Duggal et al. [7]	18	male	PO ziprasidone	a few hours	IM benztropine	Resolved in 15 min with treatment
Derinoz et al. [8]	13-18	NA	Antipsychotics	NA	NA	NA
Derinoz et al. [8]	13-18	NA	Antipsychotics	NA	NA	NA
Present Case	12	male	IV Haloperidol	24 hours	IV hydroxyzine	Resolved in a few min with treatment

PO, per oral; IM, intramuscular; IV, intravenous; NA, not available; ED, emergency department.

## References

[1] Albanese A., Bhatia K., Bressman S. B. (2013). Phenomenology and classification of dystonia: a consensus update. *Movement Disorders*.

[2] Mann S. C., Cohen M. M., Boger W. P. (1979). The danger of laryngeal dystonia. *The American Journal of Psychiatry*.

[3] Flaherty J. A., Lahmeyer H. W. (1978). Laryngeal-pharyngeal dystonia as a possible cause of asphyxia with haloperidol treatment. *Am J Psychiatry*.

[4] Goga J. K., Seidel L., Walters J. K., Khushalani S., Kaplan D. (2012). Acute laryngeal dystonia associated with aripiprazole. *Journal of Clinical Psychopharmacology*.

[5] Russell S. A., Hennes H. M., Herson K. J., Stremski E. S. (1996). Upper airway compromise in acute chlorpromazine ingestion. *The American Journal of Emergency Medicine*.

[6] Kanburoglu M. K., Derinoz O., Cizmeci M. N., Havali C. (2013). Is acute dystonia an emergency? sometimes, it really is!. *Pediatric Emergency Care*.

[7] Duggal H. S. (2007). Ziprasidone-induced acute laryngeal dystonia. *Progress in Neuro-Psychopharmacology & Biological Psychiatry*.

[8] Derinoz O., Caglar A. A. (2013). Drug-induced movement disorders in children at paediatric emergency department: 'dystonia'. *Emerg Med J*.

[9] Rupniak N., Jenner P., Marsden C. (1986). Acute dystonia induced by neuroleptic drugs. *Psychopharmacology*.

[10] Lester D. B., Rogers T. D., Blaha C. D. (2010). Acetylcholine-dopamine interactions in the pathophysiology and treatment of CNS disorders. *CNS Neuroscience & Therapeutics*.

[11] Park H. W., Kwak J. R., Lee J. S. (2017). Clinical characteristics of acute drug-induced dystonia in pediatric patients. *Clinical and Experimental Emergency Medicine*.

[12] Keepers G. A., Clappison V. J., Casey D. E. (1983). Initial anticholinergic prophylaxis for neuroleptic-induced extrapyramidal syndromes. *Archives of General Psychiatry*.

[13] Keepers G. A., Casey D. E. (1987). Prediction of neuroleptic-induced dystonia. *Journal of Clinical Psychopharmacology*.

[14] Van Harten P. N., Hoek H. W., Kahn R. S. (1999). Acute dystonia induced by drug treatment. *British Medical Journal*.

[15] Magliozzi J. R., Gillespie H., Lombrozo L., Hollister L. E. (1985). Mood alteration following oral and intravenous haloperidol and relationship to drug concentration in normal subjects. *The Journal of Clinical Pharmacology*.

[16] Volkow N. D., Gur R. C., Wang G. J. (1998). Association between decline in brain dopamine activity with age and cognitive and motor impairment in healthy individuals. *Am J Psychiatry*.

[17] ASGE Standards of Practice Committee, Early D. S., Lightdale J. R. (2018). Guidelines for sedation and anesthesia in GI endoscopy. *Gastrointest Endosc*.

[18] Rafeey M., Ghojazadeh M., Feizo Allah Zadeh H., Majidi H. (2010). Use of oral midazolam in pediatric upper gastrointestinal endoscopy. *Pediatrics International*.

[19] Motamed F., Aminpour Y., Hashemian H., Soltani A. E., Najafi M., Farahmand F. (2012). Midazolam-ketamine combination for moderate sedation in upper GI endoscopy. *Journal of Pediatric Gastroenterology and Nutrition*.

[20] Khoshoo V., Thoppil D., Landry L., Brown S., Ross G. (2003). Propofol versus midazolam plus meperidine for sedation during ambulatory esophagogastroduodenoscopy. *Journal of Pediatric Gastroenterology and Nutrition*.

[21] Brecelj J., Trop T. K., Orel R. (2012). Ketamine with and without midazolam for gastrointestinal endoscopies in children. *Journal of Pediatric Gastroenterology and Nutrition*.

[22] Paspatis G. A., Charoniti I., Manolaraki M. (2006). Synergistic sedation with oral midazolam as a premedication and intravenous propofol versus intravenous propofol alone in upper gastrointestinal endoscopies in children: a prospective, randomized study. *Journal of Pediatric Gastroenterology and Nutrition*.

[23] Disma N., Astuto M., Rizzo G. (2005). Propofol sedation with fentanyl or midazolam during oesophagogastroduodenoscopy in children. *European Journal of Anaesthesiology*.

[24] Tosun Z., Aksu R., Guler G. (2007). Propofol-ketamine vs propofol-fentanyl for sedation during pediatric upper gastrointestinal endoscopy. *Pediatric Anesthesia*.

